# Any progress in informed consenting for cancer treatment? Results from a cross sectional analysis at a comprehensive cancer center

**DOI:** 10.1007/s00432-020-03495-1

**Published:** 2021-01-09

**Authors:** Marie-Kristin Schwaegermann, Melanie Schranz, Markus Moehler, Christian Labenz, Alice Moringlane, Marcus Schmidt, Matthias Theobald, Georg Hess

**Affiliations:** 1 Department of Internal Medicine III, Comprehensive Cancer Center, University Medical Center of the Johannes Gutenberg University, Mainz, Germany; 2 Institute of Medical Biostatistics, Epidemiology and Informatics, University Medical Center of the Johannes Gutenberg University, Mainz, Germany; 3 Department of Internal Medicine I, Comprehensive Cancer Center, University Medical Center of the Johannes Gutenberg University, Mainz, Germany; 4 Department of Gynecology, Comprehensive Cancer Center, University Medical Center of the Johannes Gutenberg University, Mainz, Germany

**Keywords:** Cancer, Comprehension, Decision-making, Informed consent, Oncology, Patient–doctor relationship

## Abstract

**Purpose:**

Informed consent is required prior to any medical procedure. In the context of cancer treatment, special efforts are needed to inform cancer patients properly about treatment, potential sequelae and alternative therapies. Little is known about the effectiveness of current informed consent strategies and patients’ individual satisfaction. Given the heterogeneity in terms of age, education, sex and other factors, detailed understanding of patients’ comprehension and perception is the basis for further optimization of the informed consent process, which was the aim of the current investigation.

**Methods:**

Patients with a new cancer diagnosis and recent informed consent were asked to complete a questionnaire about satisfaction, comprehension, time management, physician–patient relationship and other items of the informed consent process. Patients were followed for 6 months and invited to complete a follow-up questionnaire.

**Results:**

In total, 89 patients completed the first questionnaire and 52 the follow-up questionnaire. Subjective understanding was assumed high, however, this did not correlate with objective understanding. Age and education were identified as influencing factors for comprehension. 85% of the patients were satisfied with the information provided. A major gap was the information on alternative therapies. Moreover, not all patients perceived the consent dialog as such, and particularly the individual treatment intention partially remained unclear for some patients.

**Conclusions:**

To ensure that informed consent is based on solid understanding, informed consenting must be patient-centered and consider the individual expectations, needs and abilities of cancer patients. Further studies are required to develop tailored informed consent strategies.

## Background

Decision-making in cancer treatment is challenging for both patient and treating oncologist. Besides full display of relevant information on therapeutic effects, risks, and potential side effects, it is important to consider patients' individual goals and expectations (Schneider et al. 2020). Hence, a comprehensive and understandable patient information prior to therapy start is a prerequisite and basis of any medical treatment. The comprehension of the underlying disease and the planned therapy is a fundamental requirement for patients with need for systemic therapy in oncology (Sato et al. 2014). A satisfactory informed consent process is of particular relevance, since oncologic treatments have an extensive impact on patients’ health and quality of life (van de Water et al. 2020). Thus, inadequate communication in cancer therapy can have a negative impact on health outcomes and impair the patient–doctor relationship (Adamson et al. 2018). In addition, patients’ level of understanding is directly linked to compliance and engagement during the therapy (Consolandi et al. 2020). In addition, providing information has a considerable impact, as patients who feel well informed tend to be more satisfied(Sato et al. 2014; Sariturk et al. 2017). Several other aspects affect patients’ satisfaction, such as the amount of time the doctor takes for the patients and the development of a doctor–patient relationship (Gericke et al. 2004; Otani et al. 2016). However, keeping the balance between medically relevant information and the actual patient interests and capabilities is a challenge in real life. Due to constantly growing medical progress, the informed consent process and thus patients’ educational material is becoming more and more extensive and complex (Larson et al. 2015). Although the use of written informed consent forms is a standard procedure, its current form is frequently forced by legal requirements, whereas it remains unclear to what extent the used forms are satisfactory and understandable for patients and some authors claim that information material on cancer therapy is often not suitable for laymen, especially in terms of readability and understandability. (Keinki et al. 2018). Additionally, a lack of understanding due to extensive length and challenging diction has often been mentioned (Nishimura et al. 2013). Moreover, finding a decent timing before therapy start as well as an appropriate duration of the informed consent process can be difficult (Murphy et al. 2016).

In this survey, we investigated whether the informed consent process for patients in daily clinical care was satisfactory and, above all, understandable at the Comprehensive Cancer Center (CCC) of the University Medical Center of the Johannes Gutenberg University Mainz, Germany. Our survey addressed the quality and the extent of the written informed consent form, the time of the informed consent talk and the associated decision time. The role of the treating physician and the physician–patient relationship were also examined in more detail.

## Methods

### Patients

In total, 100 patients with a new diagnosis of hematological or solid malignancy with need for systemic therapy were screened for this prospective study between September and December 2015 at the CCC, Mainz, Germany. Eligibility criteria included: age ≥ 18 years, written informed consent, informed consent talk within the past 2 days but no longer than 3 weeks ago and sufficient comprehension of the German language. All patients gave written consent using the standardized consent form on cytostatic system therapy, which is used by the CCC, Mainz, Germany. At study inclusion, sociodemographic factors such as school education, profession and marital status were collected.

### Questionnaire

Patients were asked to complete a questionnaire containing 40 items subdivided into the following dimensions: quality and extent of the informed consent process, time of the informed consent talk, associated decision time and patient–doctor relationship. 6 months after study inclusion patients were asked to complete a follow-up questionnaire to evaluate the informed consent process retrospectively. The first version of the questionnaire was developed in 2013 at the CCC of the University Medical Center Mainz, Germany by G. Heß and A. Moringlane, based on already published and validated questionnaires like the EORTC QLQ-INFO25 questionnaire (Arraras et al. 2011) and the Qualiskope-A questionnaire (Gericke et al. 2004). After piloting and finalizing the questionnaire, it was initially used in a study conducted at the same institution between 2013 and 2015 (“Analysis of patients’ satisfaction with informative material in the field of oncology”, A. Moringlane, 2017, unpublished doctoral thesis). We modified the initial questionnaire (changing a 10-point Likert scale to a 5-point Likert scale for a more user-friendly design) and tested its feasibility with 16 cancer patients before starting the current study at the same hospital. In total, 20 questions were designed according to a 5-point Likert scale, 8 questions were designed according to an ordinal scale, 6 questions were dichotomous and 6 questions were to be answered as a free text (e.g. “name your disease”, “which organ is mainly affected?”).

### Ethics

The study was performed in accordance with the 1964 Declaration of Helsinki and its later amendments. The study protocol was approved by the local ethics committee (approval number 837.269.15 (10037)). All study participants gave written informed consent.

## Statistical analysis

Sociodemographic characteristics (e.g. age, sex, education) and medical characteristics (e.g. therapy goal, tumor type) were analyzed with descriptive statistics. Continuous variables are presented as median and interquartile range (IQR); categorical variables are provided as absolute numbers and corresponding percentages. Patients who completed both questionnaires were compared to patients who had dropped out of the survey. This comparison was performed using the Mann–Whitney–*U* test (for continuous variables) and the Exact Fisher test (for categorical variables). Interrelations between influencing variables and response patterns were investigated by means of analysis of variance. The mean value and the standard deviation were determined and specified, when needed. For all tests, we used a 0.05 level to define statistically relevant deviation from the respective null hypothesis. Data were analyzed using IBM SPSS Statistic Version 23.0 (IBM Corp., Armonk, NY, USA).

## Results

### Patient characteristics

**Fig. 1:**
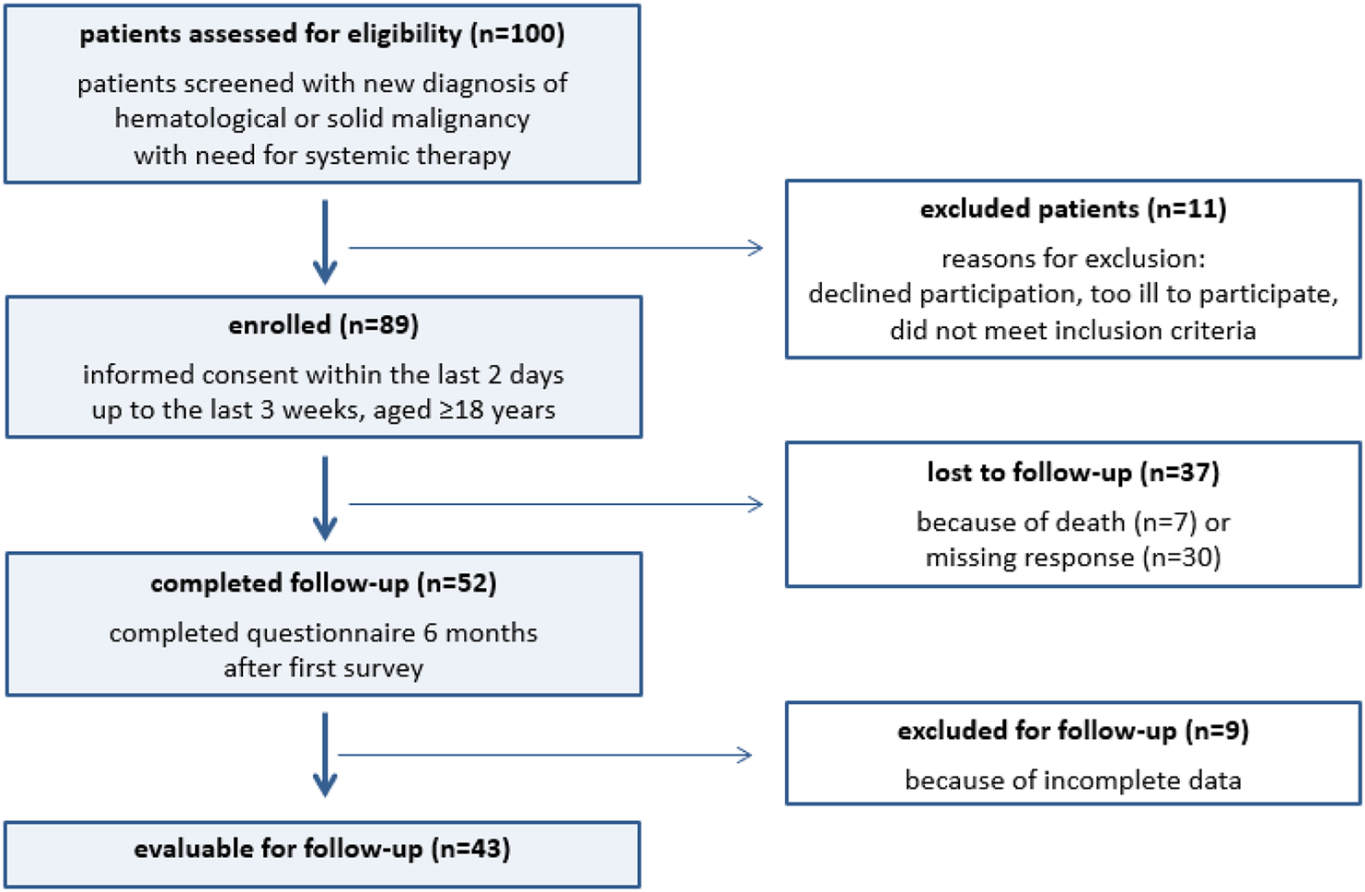
Flow diagram showing included and excluded patients as well as reasons for dropout

100 patients were screened for this study, of which 89 patients were finally enrolled and completed the questionnaire. Components of the questionnaire are displayed in Fig. 1 as well as reasons for dropout. 56% of the patients suffered from solid malignancies (cancer of the gastrointestinal tract, pancreas, lung, biliary tract, brain, skin, soft tissue, bone) and 44% of the patients suffered from hematological malignancies (acute or chronic myeloid or lymphatic leukemia, Hodgkin and non-Hodgkin lymphoma) (Table 1). 45% of the patients had a curative, 7% an adjuvant and 45% a palliative therapy goal. In 3% of the cases the treatment goal was not clearly defined at the time of study inclusion. Additional baseline characteristics of the cohort are displayed in Table 1.

**Table 1:** Baseline characteristics of the entire cohort at study inclusion

Number of patients	89 (100%)
Median age (IQR)	58,6 (18–82)
Sex	
Male	55 (62%)
Female	34 (38%)
Years of school education	
Thirteen	27 (30%)
Ten	22 (25%)
Nine	28 (32%)
Unknown	12 (13%)
Therapy goal	
Curative	39 (45%)
Adjuvant	8 (7%)
Palliative	39 (45%)
Unknown	3 (3%)

### Questionnaire

#### Duration of the informed consent process

Most patients had their informed consent talk within one week prior to therapy start (55%) and had only one single conversation with their physician (53%) (Table 2). Oral explanations usually took about 15–30 min (43%) or 30–60 min (22%). Most patients reported that the doctor took enough time to explain all relevant items. Those, whose conversation took 10 min or less, were not satisfied.

**Table 2:** Evaluation of understanding and satisfaction of the informed consent process at study initiation and 6 months later (follow-up)

Study initiation	
How much time was between the informed consent talk and therapy start? (*n* = 80)	Absolute number of patients (percentage)
Up to 1 day	17 (21%)
Up to 1 week	44 (55%)
More than 1 week	19 (24%)
How was the information presented? (*n* = 88)	
Oral only	24 (27%)
Only by written material	0 (0%)
Both orally and by written material	64 (73%)
Were different therapy options discussed? (*n* = 89)	
Yes	46 (52%)
No	39 (43%)
I do not remember	4 (5%)
How many conversations did you have with your doctor before signing the informed consent form? (*n* = 89)	
One	47 (53%)
Two	26 (29%)
More than two	16 (18%)
I had enough time to overthink my agreement to therapy (*n* = 84)	
Fully agree	38 (45%)
Rather agree	27 (32%)
Partially	10 (12%)
Rather not agree	6 (7%)
Do not agree at all	3 (4%)
How long did the oral explanation about the planned therapy take? (*n* = 89)	
Up to 5 min	1 (1%)
Up to 10 min	10 (12%)
Up to 15 min	12 (13%)
15–30 min	38 (43%)
30–60 min	20 (22%)
More than 60 min	8 (9%)
How much time did you need to read the informed consent material? (*n* = 75)	
Up to 15 min	28 (38%)
15–30 min	31 (41%)
30–60 min	4 (5%)
More than 60 min	4 (5%)
I did not read it	8 (11%)
I read the informed consent material completely (*n* = 89)	
Fully agree	30 (34%)
Rather agree	23 (26%)
Partially	20 (22%)
Rather not agree	9 (10%)
Do not agree at all	7 (8%)
The aim of the therapy was clearly discussed during the informed consent process (*n* = 88)	
Fully agree	35 (40%)
Rather agree	31 (35%)
Partially	16 (18%)
Rather not agree	4 (5%)
Do not agree at all	2 (2%)
The consent form was easy to understand (*n* = 84)	
Fully agree	29 (35%)
Rather agree	31 (37%)
Partially	18 (21%)
Rather not agree	4 (5%)
Do not agree at all	2 (2%)
I had to ask a lot of questions to understand the informed consent form (*n* = 82)	
Fully agree	2 (2%)
Rather agree	6 (7%)
Partially	19 (23%)
Rather not agree	35 (43%)
Do not agree at all	20 (25%)
There were many incomprehensible words in the text (*n* = 82)	
Fully agree	3 (3%)
Rather agree	8 (10%)
Partially	28 (34%)
Rather not agree	26 (32%)
Do not agree at all	17 (21%)
I just wanted to sign the informed consent form (*n* = 87)	
Fully agree	5 (6%)
Rather agree	20 (23%)
Partially	17 (19%)
Rather not agree	20 (23%)
Do not agree at all	25 (29%)
I felt reassured after the informed consent (*n* = 88)	
Fully agree	11 (12.5%)
Rather agree	32 (36%)
Partially	30 (34%)
Rather not agree	11 (12.5%)
Do not agree at all	4 (5%)
Chances and risks were well presented (*n* = 89)	
Fully agree	31 (35%)
Rather agree	36 (40%)
Partially	16 (18%)
Rather not agree	6 (7%)
Do not agree at all	0 (0%)
It was important to me to be informed about all side effects (*n* = 88)	
Fully agree	53 (60%)
Rather agree	21 (24%)
Partially	11 (13%)
Rather not agree	2 (2%)
Do not agree at all	1 (1%)
The doctor took enough time to explain everything (*n* = 89)	
Fully agree	51 (57%)
Rather agree	25 (28%)
Partially	9 (10%)
Rather not agree	3 (4%)
Do not agree at all	1 (1%)
Follow up	
Retrospectively, the informed consent displayed things decently (*n* = 43)	
Fully agree	22 (52%)
Rather agree	16 (37%)
Partially	3 (7%)
Rather not agree	1 (2%)
Do not agree at all	1 (2%)
Retrospectively, I am satisfied with the information I received (*n* = 43)	
Fully agree	20 (47%)
Rather agree	19 (44%)
Partially	3 (7%)
Rather not agree	1 (2%)
Do not agree at all	0 (0%)
I would have liked more information after all (*n* = 43)	
Fully agree	3 (7%)
Rather agree	4 (9%)
Partially	11 (26%)
Rather not agree	15 (35%)
Do not agree at all	10 (23%)
The therapy proceeded in the same way as the informed consent talk anticipated (*n* = 43)	
Fully agree	19 (44%)
Rather agree	17 (40%)
Partially	6 (14%)
Rather not agree	1 (2%)
Do not agree at all	0 (0%)

Regarding the time needed to read the information material, most patients took 15–30 min (41%) or up to 15 min (38%). 11.5% did not read the written informed consent form at all. Elderly patients estimated their required reading time quite long, whereas young patients indicated a rather short reading time.

45% of the patients were satisfied with the given time between the informed consent talk and the therapy start, 12% were partially satisfied and 11% stated that the time was not sufficient.

Patients were asked about their individual satisfaction with the time window between the informed consent talk and the actual therapy start. The feeling of satisfaction was present in 78% of the patients when the talk was two weeks prior to the therapy start and 40% when the gap was only one week.

### Comprehension

Most patients affirmed that the doctor explained their treatment goal very well (40%) or mostly well (35%). However, for 18% of the patients the treatment goal remained partially unclear and altogether 7% of the patients did not know the treatment goal at all. Most patients in this group received palliative care.

43% of patients stated that no alternative therapy options were discussed. However, the statement "chances and risks of the therapy were presented well" was affirmed by 75% of patients.

Most patients considered the written informed consent form as decently understandable (71%). 30% of the patients stated that the consent form partially contained difficult words. By comparing mean values, we found that patients with low school formation indicated more incomprehensible words than patients with at least 10 or 13 years of school formation (mean value low school formation = 2,92 vs. mean value middle school/ high school = 2,00). Using a Kruskal–Wallis test we examined whether the different educational levels had an impact on the incomprehensibility of the text. A striking difference was found between the groups (χ2(3) = 11,655, *p* = 0.003). Assuming a significance level of 5%, the difference is significant. Using *U* tests (Mann–Whitney) we tested the different educational levels in pairs regarding the comprehensibility. At a significance level of 5%, patients with lowest school education indicated significantly more incomprehensible words in the text than patients with middle school (*U* = 140, *p* = 0.005) and high school formation (*U* = 156.5, *p* = 0.003). Further Kruskal–Wallis tests were used to examine whether differences in patients’ educational level correlate with the amount of questions that were asked during the informed consent talk. This hypothesis could not be proven (χ2(2) = 2.332, *p* = 0.312). Moreover, the length of the informed consent talk did not influence patients’ subjective feeling of having been well informed.

### Patients’ attitude towards consent

Patients' perception of the informed consent process differed quite much. The statement "I just wanted to sign the informed consent without further information" was affirmed by about one third of the patients (29%) and negated by half of the patients (52%). Moreover, patients who stated that they just wanted to sign did not read the form completely either (*r* = −0.376, *p* < 0.001, correlation according to Spearman).

Most relevant information for the patients during the informed consent process were (in descending order) information about the disease and its background, about the proposed therapy, about alternative therapies and about side effects. Information about time sequences, accompanying exams, contraception and interaction of medication were considered less important.

## Follow-up evaluation

52 of the initially 89 patients completed the follow-up questionnaire six months after therapy start. Of the 37 dropouts, 7 patients died within this period. Differences between participants and dropouts were found both in demographic and medical variables. Among the patients who completed the follow-up questionnaire were significantly more patients with a curative therapy goal (*p* < 0.001). Among the patients who dropped out were significantly more patients with a low school formation (*p* 0.020). No differences were found regarding sex and age.

Most patients stated that retrospectively the informed consent process illustrated things well and that they were satisfied with the information given (Table 2). One fourth lacked information on some parts, 16% lacked information in general. Looking at subgroups, a correlation with school education was found. Patients who attended high school were more likely to ask for more information. Noticeably, only 40% of patients were satisfied with information on alternative therapies.

## Discussion

In this study, we investigated patient’s individual comprehension and satisfaction of current informed consent procedures. We could clearly demonstrate in our series that age and a lower education level have an impact on patients’ comprehension, which in turn correlates with individual satisfaction. Prior studies already indicated that patients' understanding decreases significantly with increasing age and a lower level of school education (Casarett et al. 2003; Sanchini et al. 2013; Sherlock and Brownie 2014), which is in line with our findings. In contrast to other studies, gender did not influence the understanding of the consent process in our current study (Wagner et al. 2019).

When evaluating the effectiveness of an informed consent process a distinction between subjective and objective understanding is critical (Joffe et al. 2001). While the subjective feeling of having been well informed was high in this current survey, this did not result in a high degree of objective understanding. As an example, a substantial proportion of patients was not able to properly answer the question about their individual diagnosis or their distinct treatment aims. Some patients only mentioned the generic term "cancer" at this point, others did not answer the question at all, which illustrates a lack of understanding.

Some authors claim that the spelling style of informed consent material is too complex and often exceed patients' reading comprehension (Larson et al. 2015). In our survey, we could in part find the same observation, as 13.5% of patients stated that the text contained many incomprehensible words and 34% experienced partial difficulties in understanding. The observed discrepancy between reported subjective satisfaction and difficulties in comprehension can be explained with the effect of social desirability. Older series have demonstrated a higher rate of difficulties, which in turn may reflect improvements in the comprehensibility of current materials.

Data indicate that patients often do not read the consent material at all (D’Souza et al. 2015), which applies for 11% of patients in this survey. About 40% of patients needed a maximum of 15 min to read the informed consent form, which—in light of the volume, complexity and scope—raises skepticism about complete understanding.

Most patients were satisfied with the timing of the informed consent talk before therapy initiation. Those who were dissatisfied either consented on the very same day of therapy start or more than 2 weeks before, indicating that too short and too long intervals between the informed consent talk and therapy start do not meet patients' needs. Overall, most patients indicated that their doctor took enough time to explain everything. In contrast, patients whose conversation only took up to 10 min were only 50% satisfied.

About one third of the patients stated that they consented only verbally, which is questionable since handing out a copy of the signed informed consent form to the patient is a standard procedure. Thus, it can be assumed that not all patients perceive the informed consent talk as their main informative talk. To avoid this misconception, doctors should introduce the purpose of the talk right at the beginning, e.g. by emphasizing that both oral and written informed consent will take place in the next 30 min.

Only few patients had a sense of reverence (and thus restraint) towards the doctor as described in the literature (Hall et al. 2012). Group-specific differences were found regarding school education: patients with low school education were more likely to feel obligated (12.5%) than patients with high school (8%) or intermediate school education (4.5%). Whereas a few decades ago a paternalistic attitude regarding therapy decisions prevailed, patients today are aware of their right to self-determination and freedom of choice (Krishnan and Kasthuri 2007).

Patients were generally satisfied with the information provided, also during the follow-up. However, retrospectively, dissatisfaction was noted regarding information about alternative therapies: 40% stated that they did not feel well informed in this regard. Comparing this to the first survey, it is striking that initially only 29% of the patients stated that information on alternative therapies was important to them. Thus, a change in the need for information during cancer treatment can be assumed, especially for those who do not respond to cancer therapy. A central insight of this survey is that even if there is no alternative therapy option, this information should also be communicated to improve patient satisfaction. 30% of patients were only partially or not satisfied with the display of opportunities and risks of the planned therapy. To discuss risk, van de Water et al. suggest that clinicians should not only use words when describing risks but at least also use some form of numbers or visualization (van de Water et al. 2020).

Still, the influence of emotions should not be underestimated here. According to Visser et al. patients often cannot remember relevant topics of their informed consent talk due to fear, agitation or pain.(D'Souza et al. 2015; Sariturk et al. 2017; Visser et al. 2017). This is of pivotal relevance when the doctor must break bad news as e.g. the lack of curative treatment options. Although in this survey most patients knew their treatment goal after the informed consent talk, for 20% of the patients it was not or only partially clear. Strikingly, most of them had a palliative therapy goal.

To objectify the level of patient understanding in future projects, the use of validated questionnaires as the EORTC QLQ-INFO25 questionnaire is promising (Arraras et al. 2011). To increase patients satisfaction and the level of understanding during the informed consent process, potential new techniques as conducting structured interviews, using “feedback techniques” or audiovisual tools such as tablets with PowerPoint presentations containing the most relevant information should be considered (Sanchini 2013; Glaser et al. 2020). So called "patient coaches", as sometimes established in clinical trials, could help to provide information instead of the doctor. Handing out additional informative material to optimize patient understanding seems less important, since only 60% of patients read the written material completely.

Finally, with increasing demographic aging, informed consent forms need to be adapted in terms of readability. An optimized, age-adapted version should have a larger font size and stronger contrasts, as well as simpler and more concise language (Spellecy et al. 2018; Glaser et al. 2020)—not only for elderly patients. The impact of these features on patients’ satisfaction and understanding of the informed consent process have to be evaluated in future trials.

## Study limitations

Our study has some limitations that have to be acknowledged. First, the questionnaire used was developed, piloted, and validated at our own hospital, but has not been validated nationally or internationally. Since the same questionnaire was used for two studies at the same hospital, we believe that its feasibility is sufficiently established. Our findings may only hold true for German patients and may not be generalized for patients from other countries. Second, there may be some room for sample bias between the initial cohort and the follow-up cohort. Patients of the follow-up cohort differed significantly from the initial cohort regarding the frequency of curative therapy goals and school education.

## Clinical implications

With increasingly complex treatments available in oncology, consenting is more than a simple explanation of side effects of a therapy. It covers a wide range from the selection of the right treatment out of a variety of options in the light of therapeutic chances and potential harms of treatment. This complexity combined with the individuality of each patient requires a non-standardized but specified consenting process. The findings of this study underline this necessity, but at the same time highlight the difficulty to implement this into clinical routine. Accordingly, the translation of findings like ours into an optimized, patient-centered portfolio will only succeed through interdisciplinary collaboration, to improve patient understanding and satisfaction.

## Conclusions

In summary, patients felt subjectively well informed, however, this did not always correlate with objective understanding. Most patients were satisfied with the information provided, regardless of whether the informed consent talk took place on the same day of therapy start or up to two weeks before, implying that the way of presenting information is more important than the timing. The length of the informed consent process did not impact satisfaction or understanding. Striking findings are that patients retrospectively often lacked information on alternative therapies. Also, not all patients perceived the educational talk as such, and the therapy intention was partially unclear.

Our data show that the informed consent process even at a specialized large academic center is not satisfying all patients’ needs. Patient-doctor-communication must be optimized with focus on strategies to present information in a more concrete and simple way. To meet cancer patients’ individual expectations and needs, the emphasis should be on patient-centered approaches. For those patients, who do not want to know every detail of their disease and therapy and who prefer basic information, a concise summary accompanied by meaningful graphics could be a solution. For those who prefer more details, background knowledge could be provided in form of an application for smart phones or tablets or in an individualized portfolio. There certainly is no ‘one-size-fits-all’ approach to informed consent in cancer treatment. That is why we will further investigate patient-centered approaches in upcoming research projects to develop optimized informed consent strategies.

## Data Availability

The data that support the findings of this study are available from the corresponding author upon reasonable request.
